# Effect of Cathodal Transcranial Direct Current Stimulation on a Child with Involuntary Movement after Hypoxic Encephalopathy

**DOI:** 10.1155/2018/8934253

**Published:** 2018-03-25

**Authors:** Mayumi Nagai, Naofumi Tanaka, Yutaka Oouchida, Shin-Ichi Izumi

**Affiliations:** ^1^Department of Physical Medicine and Rehabilitation, Tohoku University Graduate School of Medicine, 2-1 Seiryo-cho, Aoba-ku, Sendai 980-8575, Japan; ^2^Department of Rehabilitation Faculty of Medical Science and Welfare, Tohoku Bunka Gakuen University, 6-45-1 Kunimi, Aoba-ku, Sendai 981-8551, Japan; ^3^Department of Physical Medicine and Rehabilitation, Tohoku University Graduate School of Biomedical Engineering, 2-1 Seiryo-cho, Aoba-ku, Sendai 980-8575, Japan

## Abstract

The aim of the study was to investigate the effect of cathodal transcranial direct current stimulation to the supplementary motor area to inhibit involuntary movements of a child. An 8-year-old boy who developed hypoxic encephalopathy after asphyxia at the age of 2 had difficulty in remaining standing without support because of involuntary movements. He was instructed to remain standing with his plastic ankle-foot orthosis for 10 s at three time points by leaning forward with his forearms on a desk. He received cathodal or sham transcranial direct current stimulation to the supplementary motor area at 1 mA for 10 min. Involuntary movements during standing were measured using an accelerometer attached to his forehead. The low-frequency power of involuntary movements during cathodal transcranial direct current stimulation significantly decreased compared with that during sham stimulation. No adverse effects were observed. Involuntary movement reduction by cathodal stimulation to supplementary motor areas suggests that stimulations modulated the corticobasal ganglia motor circuit. Cathodal stimulation to supplementary motor areas may be effective for reducing involuntary movements and may be safely applied to children with movement disorders.

## 1. Introduction

Involuntary movements, including those of hyperkinetic nature, hinder motor development in children. Hyperkinetic movement disorders are characterized by excessive and abnormal involuntary movements and include myoclonus, dystonia, and chorea. They are considered to mainly occur because of the impairment of basal ganglia (BG), reported to be closely linked to cortical brain areas such as the supplementary motor area (SMA) and primary motor cortex (M1) [1]. Certain nuclei in BG and motor-related cortical areas comprise the corticobasal ganglia (CBG) motor network [2], playing an important role in adjusting voluntary movements by inhibiting involuntary movements. The CBG motor circuit has three pathways. One is the direct pathway that is involved in initiating body movements by increasing thalamic excitability. The other two are the indirect and hyperdirect pathways, which are involved in inhibiting body movements by decreasing thalamic activity. The hyperdirect pathway is involved in motor preparation prior to motor execution, while the indirect pathway engages in movement cessation. A previous study reported that the input of the direct pathway was markedly increased compared with the other two pathways in hyperkinetic movement disorders [3]. This evidence suggests that the hyperactivity of the direct pathway in the CBG motor network is a crucial factor in hyperkinetic movement disorders.

In clinical situations, pharmacological therapy is frequently used for children with hyperkinetic movement disorders, although it is associated with a risk of serious adverse effects such as addiction and dependency [4]. Therefore, a safer treatment option with fewer adverse effects is required for children with hyperkinetic movement disorders. Transcranial direct current stimulation (tDCS) has recently drawn attention as a safe noninvasive method for modulating brain activity. The DCS supplies a weak electrical current of 1 or 2 mA to the brain via two surface electrodes on the head skin to facilitate (anodal stimulation) or inhibit (cathodal stimulation) cortical excitability of the stimulated brain area [5]. No adverse effects using tDCS in adult participants have been previously reported [6]. In addition, a previous study showed that cathodal tDCS to M1 safely improved dystonia symptoms in children [7]. Thus, tDCS has a great potential to be a safe and effective method of brain stimulation for children with movement disorders.

Here, we report a case of an 8-year-old boy with an abnormal movement of head and neck, a hyperkinetic movement disorder, following brain damage. We examined whether cathodal tDCS to SMA suppressed the abnormal excitability of SMA to reduce the activity of the CBG motor circuits, resulting in the inhibition of involuntary movements.

## 2. Case Report

**Figure 1:**
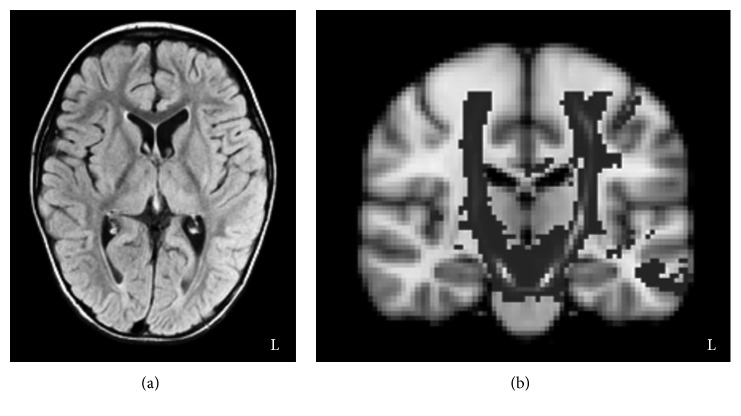
Brain magnetic resonance and fiber tracking images. (a) A brain magnetic resonance image at age 6 showing the mild degree of cerebral atrophy. (b) A fiber tracking image reconstructed corticospinal tract in both hemispheres.

We present a case of an 8-year-old boy with involuntary movements of his limbs and trunk. He had hypoxic encephalopathy because of cardiopulmonary arrest after choking on food at the age of 2. He lost consciousness for 18 min owing to the cardiopulmonary arrest and was treated with hypothermia and steroid pulse therapies at our hospital for 3 months. He was then transferred to another hospital to undergo rehabilitation and returned home after 5.5 months. He underwent outpatient rehabilitation at our hospital since the age of 6. Results of the auditory reception subtest of the Illinois Test of Psycholinguistic Abilities indicated that he had an intellectual level of 5 years and 8 months; that is, he was normal in intellectual level. Although he had severe dysarthria, he was able to communicate using gestures or a communication board. Magnetic resonance (MR) imaging of his brain at the age of 6 showed a mild degree of cerebral atrophy with no clear BG lesion (Figure 1(a)). Fiber tractography using diffusion tensor imaging revealed no serious damage to the corticospinal tracts on either side (Figure 1(b)). His family history, perinatal period, developmental history, and past medical history were all normal. In his clinical assessments, manual muscle testing scores of the upper extremities, trunk, and lower extremities were 4/5, 2/5, and 2/5, respectively, with no side-to-side differences. Although his muscle tone and somatosensory function were normal, he was unable to maintain his posture or stabilize his extremities because of involuntary movement that involved the whole body. In addition, his involuntary movement was exacerbated before he initiated a voluntary movement. In daily life, he required a trunk belt to sit on a chair, and he could not stand without the support for his weight with both hands.

**Figure 2:**
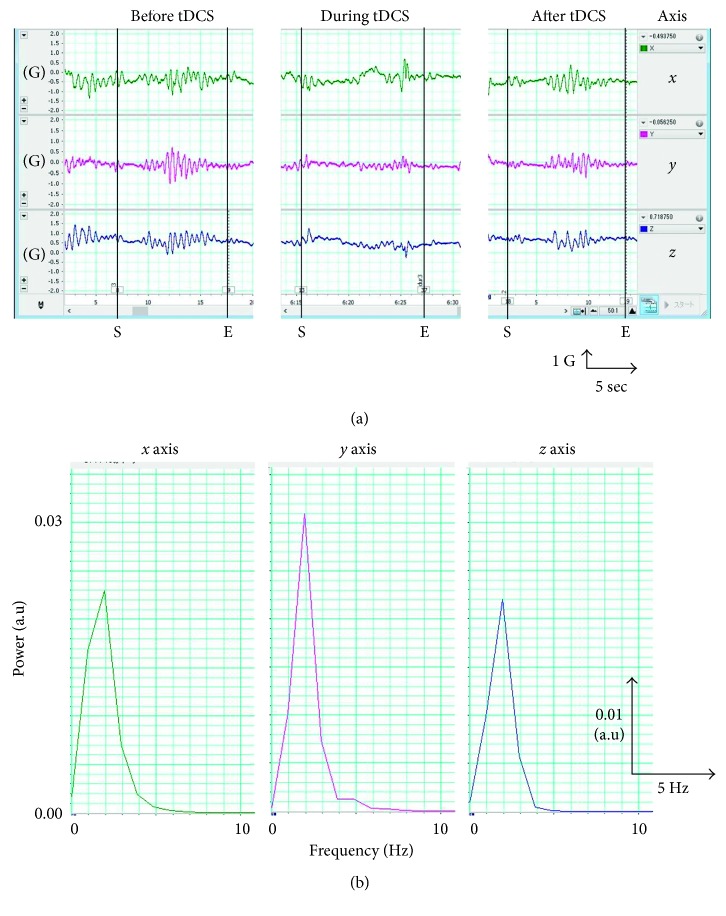
Typical acceleration data of head motion in a single trial. (a) The raw data along the *x*-axis (green), *y*-axis (pink), and *z*-axis (blue) before, during, and after tDCS. (b) The power spectrum of *x*-axis (green), *y*-axis (pink), and *z*-axis (blue). S and E indicate the start and end of the trial, respectively.

The patient was instructed to remain standing with his plastic ankle-foot orthosis for 10 s, leaning forward on a desk on his forearms. Movement of his head during standing was measured using an accelerometer attached to the glabella for 10 s at three time points: before, during, and after tDCS. Acceleration data were recorded at a sampling rate of 1 kHz using an analog-to-digital converter (Power Lab 16/35; AD Instruments, Aichi, Japan) (Figure 2(a)) and were analyzed using specialized software (Lab Chart 7; AD Instruments, Aichi, Japan) to calculate and sum the power spectra within 1–5 Hz for each axis on three dimensions (*x*-, *y*-, and *z*-axis) (Figure 2(b)). We compared the ratios of power spectrum data (% power) during and after tDCS and divided this by the data prior to tDCS.

For the effective stimulation of tDCS, before this experiment we had already administered both anodal and cathodal stimulation to SMA and M1 of the patient and selected the cathodal stimulation to SMA from the other stimulations. Cathodal or sham tDCS was transcranially delivered to SMA at 1 mA for 10 min using a DC stimulator (neuroConn GmbH, Ilmenau, Germany) with 7 × 5 cm electrodes. Each stimulation session was conducted for 3 days, at least 2 days apart. The cathodal electrode was placed over SMA, identified as 2 cm anterior to Cz in the International 10/20 EEG System, and the anodal electrode was placed over the left supraorbital region. In the stimulation period, the participant started the task 2 min after stimulation began. During stimulation, he was asked to keep standing for 10 s at three time points without interruption.

The safety of tDCS was assessed by interviewing the patient and his mother, as well as through the examiner's observations. The patient was monitored and frequently asked whether he experienced pain or discomfort during and after tDCS. Before and after tDCS sessions, the experimenter carefully checked the skin where the electrodes were placed. The study protocol was approved by the Ethics Committee of Tohoku University Graduate School of Medicine. The patient and his parent gave informed written consent.

Percent power data were analyzed via two-way analysis of variance (ANOVA) with the factors “stimulation” (cathodal stimulation and sham stimulation) and “time” (before, during, and after tDCS). In post hoc analysis, multiple comparison was performed using the Bonferroni correction. The level of significance was set at *p* < 0.05. All analyses were performed using SPSS for Windows (version 20.0; IBM, Armonk, NY, USA).

**Figure 3:**
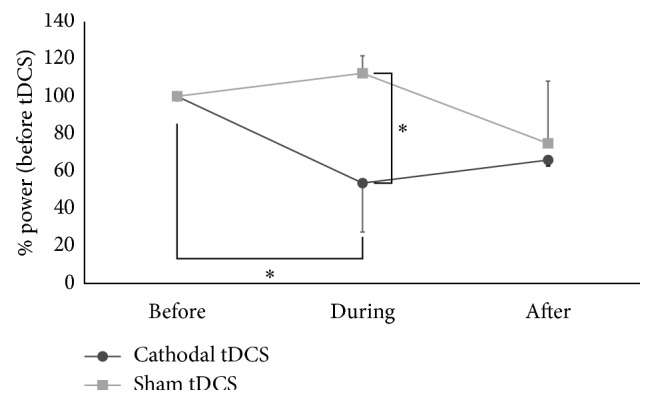
Results of the ratios of the power spectrum data. Mean ratios of the power spectrum data for 3 days before, during, and after the cathodal tDCS (black circles) and the sham tDCS (gray circles) to those before the cathodal tDCS and the sham tDCS, respectively. Asterisk indicates *p* < 0.05. Error bars indicate standard deviation.

Figure 3 showed results of % power data. The two-way ANOVA showed main effects of “stimulation” (*F* = 7.3, *p* < 0.05) and “time” (*F* = 4.2, *p* < 0.05). There was a statistically significant interaction of “stimulation” × “time” (*F* = 4.8, *p* < 0.05). Specifically, the % power during tDCS was significantly reduced compared with that during sham stimulation. The % power during tDCS was also significantly reduced, compared with that before tDCS but not with that during sham stimulation. Post hoc analysis for the factor “stimulation” showed that % power during tDCS was decreased, compared with that during sham stimulation. Post hoc analysis for the factor “time” showed that % power during tDCS was significantly decreased, compared with that before tDCS. The study sessions were completed with no adverse effects.

## 3. Discussion

We examined whether cathodal tDCS to SMA could inhibit rapid involuntary movements with cyclic patterns in an 8-year-old boy with hyperkinetic disorder. Results demonstrated that cathodal tDCS to SMA decreased involuntary movements of the head and neck during standing, as measured using an accelerometer attached to the forehead. Given that we observed no issues or adverse effects from tDCS, we conclude that tDCS is potentially a safe method for treating children with involuntary movement disorders.

Although the exact mechanism of reducing involuntary movements by tDCS is unknown, there are several possibilities for explaining this effect. A possible hypothesis is that tDCS to SMA can modulate abnormal excitation of the CBG network by inhibiting SMA activity. In turn, this could lead to decreased activity of the striatum, which inhibits the inhibitory output from the BG network to the thalamus modulating the activity of the corticospinal tract [2].

The CBG motor network plays an important role in modulating voluntary movement and inhibiting involuntary movement during motor execution. In this study, cathodal tDCS to SMA could inhibit SMA activity, causing to decrease the abnormal activity of the BG. Furthermore, this would inhibit the activity of the thalamus via the striatum and internal segment of the globus pallidus, resulting in decreased excitability of M1. In summary, the cathodal tDCS to SMA normalized the activity of the CBG motor circuit. In the present study, there is a possibility that the tDCS stimulated the M1 directly because the size of the electrodes used was 7 × 5 cm. This direct stimulation to the M1 area may decrease the abnormal involuntary movements of his body by inhibiting the activity of the M1 area that innervates the movement of the foot and trunk.

There was another possibility that the patient's abnormal involuntary movement was caused by Lance–Adams syndrome (LAS), with involuntary movements developed after hypoxic encephalopathy. Although no obvious lesion was detected in MR images, his involuntary movements first appeared after cardiopulmonary arrest. Previous studies reported that LAS is responsible for the hyperactivity of the thalamocortical pathway, which is modulating by the motor-related cortical areas such as SMA and M1 [8, 9]. Even if his abnormal involuntary movement was derived from LAS, tDCS to SMA could modulate the hyperactivity of the thalamocortical pathway, resulting in decreasing involuntary movements associated with the M1 activity.

Although some previous studies reported that tDCS induced adverse effects such as tingling, burning under the electrodes, sensation-like pain, and headache [6], no adverse effects were observed in our study. Our findings suggest that tDCS is a safe treatment method for children with involuntary movement disorders. A limitation of this study was that the reduction of the abnormal involuntary movements by tDCS was only the short-lasting effects. Further studies must confirm the long-lasting effects of tDCS on hyperkinetic movement disorders in children in order to apply tDCS to clinical practice.

## References

[1] Middleton F. A., Strick P. L. (2000). Basal ganglia and cerebellar loops: motor and cognitive circuits. *Brain Research Reviews*.

[2] Alexander G. E., DeLong M. R., Strick P. L. (1986). Parallel organization of functionally segregated circuits linking basal ganglia and cortex. *Annual Review of Neuroscience*.

[3] Nishibayashi H., Ogura M., Kakishita K. (2011). Cortically evoked responses of human pallidal neurons recorded during stereotactic neurosurgery. *Movement Disorders*.

[4] Hanayama K. (2004). Effects of physical therapy in children with cerebral palsy. *Journal of Clinical Rehabilitation*.

[5] Nitsche M. A., Paulus W. (2001). Sustained excitability elevations induced by transcranial DC motor cortex stimulation in humans. *Neurology*.

[6] Poreisz C., Boros K., Antal A., Paulus W. (2007). Safety aspects of transcranial direct current stimulation concerning healthy subjects and patients. *Brain Research Bulletin*.

[7] Young S. J., Bertucco M., Sanger T. D. (2014). Cathodal transcranial direct current stimulation in children with dystonia: a sham-controlled study. *Journal of Child Neurology*.

[8] Lance J. W., Adams R. D. (1963). The syndrome of intention or action myoclonus as a sequel to hypoxic encephalopathy. *Brain*.

[9] DeLisa J. A., Stolov W. C., Troupin A. S. (1979). Action myoclonus following acute cerebral anoxia. *Archives of Physical Medicine and Rehabilitation*.

